# Neurosteroid involvement in threatened preterm labour

**DOI:** 10.1002/edm2.216

**Published:** 2020-12-10

**Authors:** Sahruh Turkmen, Torbjörn Bäckström, Yvonne Kangas Flodin, Marie Bixo

**Affiliations:** ^1^ Sundsvalls Research Unit Department of Clinical Sciences, Obstetrics and Gynaecology Umeå University Sundsvall Sweden

**Keywords:** allopregnanolone, GABA_A_ receptor, oxytocin, progesterone, saccadic eye velocity

## Abstract

**Introduction:**

The neurosteroid allopregnanolone modulates oxytocin expression in the brain, and its effects arise from its action on the GABA_A_ receptor. Whether neurosteroid levels and the function of the GABA_A_ receptor are involved in the risk of preterm labour in pregnant women is unknown.

**Methods:**

Pregnant women with (*n* = 16) or without (*n* = 20) threatened preterm labour (TPL) in gestational week 33 + 6 days to 37 + 0 days were studied prospectively with procedures including foetal heart rate monitoring, vaginal examination, ultrasound examination and blood tests to determine allopregnanolone, progesterone and oxytocin levels. The GABA_A_ receptor function in both groups was measured with a saccadic eye velocity test (SEVT).

**Results:**

Plasma oxytocin levels were higher in the TPL group than in the control group (41.5 vs. 37.0 pmol/L, respectively, *p* = .021). Although the allopregnanolone and progesterone levels in both groups did not differ, there was a negative association between blood oxytocin and allopregnanolone (as predictor) levels in the TPL group (B: −3.2, 95% confidence interval (CI): −5.5 to −0.9, *p* = .012). As a predictor of TPL, progesterone was associated with cervix maturity (odds ratio: 1.02, 95% CI: 1.00–1.04, *p* = .038). SEVT showed that the women in both groups had similar GABA_A_ receptor functions. In both groups, body mass index correlated with peak saccadic eye velocity (*r* = .34, *p* = .044) and negatively with allopregnanolone (*r* = −.41, *p* = .013).

**Conclusions:**

Neurosteroid levels were unchanged in the peripheral blood of women with TPL, despite the increase in available oxytocin. Although the function of the GABA_A_ receptor was unchanged in women with TPL, to ensure reliable results, saccadic eye velocity should be investigated during a challenge test with a GABA_A_ receptor agonist.

## Graphical abstract

The GABAergic neurosteroid allopregnanolone inhibits oxytocin synthesis during pregnancy, and in women with threatened preterm labour, the levels of the allopregnanolone and progesterone in the peripheral blood and GABAA receptor function were unchanged, despite the increase in available oxytocin, but an association between allopregnanolone and oxytocin levels may suggest a central mechanism in which allopregnanolone controls the peripheral oxytocin levels.

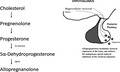

## 1 BACKGROUND

Preterm birth, defined as birth before 37 weeks of gestation, is a significant public health problem. It affects 5%–18% of pregnancies (5%–6% in Sweden) and is the leading cause of neonatal mortality, as well as serious neonatal morbidity.^1^, ^2^

**Figure 1:**
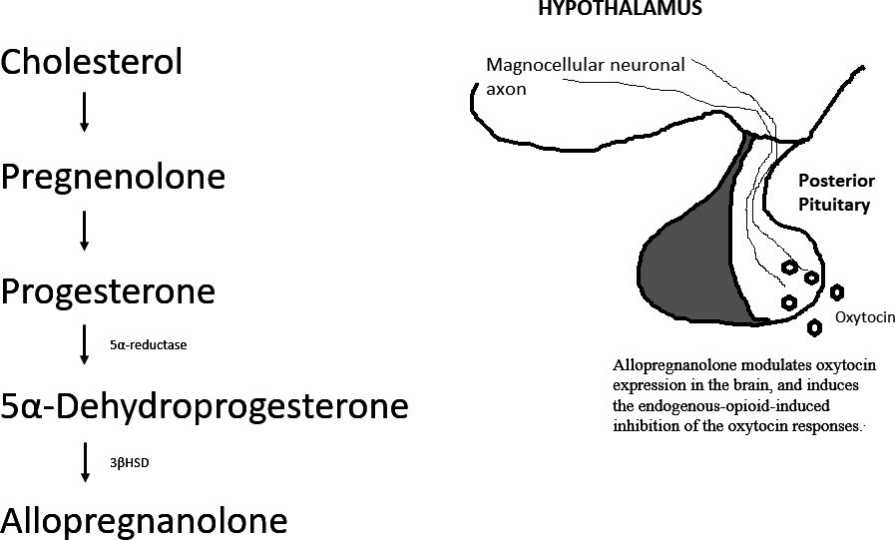
Allopregnanolone synthesis pathway. 3βHSD, 3beta‐hydroxysteroid dehydrogenase

Two‐thirds of preterm births occur after the spontaneous onset of labour.^3^ The parturition process depends on the mechanism by which a quiescent myometrium enters a contractile state and is accompanied by a shift in signalling from anti‐inflammatory to pro‐inflammatory pathways.^4^ Early animal studies have shown that the uterine myometrium is held in a state of quiescence by progesterone and that normal labour is initiated by a fall in progesterone levels.^5^ Several subsequent studies, although conflicting, have assessed the efficacy of progesterone in preventing uterine contractions and preterm delivery,^6^ and it is now widely recognized that progesterone plays a major role in maintaining uterine quiescence in human pregnancy.^7^ During human pregnancy, progesterone concentrations gradually increase to maintain uterine quiescence and prevent abortion or premature labour,^8^ but this is also accompanied by increasing levels of the neurosteroid allopregnanolone, a metabolite of progesterone^9^, ^10^ (Figure 1). In the serum and umbilical cord, progesterone concentrations are higher during normal, spontaneous or oxytocin‐induced delivery, compared with those in oxytocin‐resistant dystocia and in pregnant women whose delivery has not yet started.^11^ These inconsistent results have prompting further studies of the onset and effectiveness of the normal delivery progress.

Oxytocin is a peptide hormone normally produced in the hypothalamus and released by the posterior pituitary.^12^ It plays an important role in promoting uterine contractions during parturition and in stimulating the ejection of milk during lactation. Oxytocin and allopregnanolone are both secreted in response to stress,^13^ but the premature activation of oxytocin secretion by stressors in late pregnancy is hindered by the activation of a central endogenous opioid mechanism.^14^ For example, acute morphine treatment inhibits the oxytocin release stimulated by parturition,^15^ and when the actions of endogenous opioids are antagonized with naloxone, the oxytocin response to the immune stressor interleukin‐1β is potentiated.^14^ When the pregnancy levels of progesterone were simulated in virgin rats, opioid inhibition of the oxytocin response to stress was induced.^16^ These activities may be attributed to increased production of the neurosteroid allopregnanolone, which is abundant in the brain during late pregnancy and reduces stress‐induced hypothalamus‐pituitary‐adrenal axis activity.^9^, ^17^ Allopregnanolone is produced from progesterone by the sequential action of the enzymes 5α‐reductase and 3α‐hydroxysteroid dehydrogenase, and in late pregnancy, the amounts of both enzymes increase in brain regions known to be critical in regulating the oxytocin responses to stress.^17^, ^18^ Changes in serum allopregnanolone levels in healthy women are not fully dependent on variations in progesterone.^19^ Several studies have shown that the neurosteroid allopregnanolone modulates oxytocin expression in the brain and induces the endogenous opioid‐induced inhibition of the oxytocin responses.^20^, ^21^ However, the mechanism by which allopregnanolone induces inhibitory opioid tone is still unclear, although it may involve an interaction with the GABA_A_ receptor. Allopregnanolone acts as an allosteric modulator of the GABA_A_ receptor and regulates oxytocin gene expression in late pregnancy.^20^, ^22^ When allopregnanolone acts centrally, it induces an inhibitory opioid mechanism that maintains the quiescence of the magnocellular oxytocin system. Morphine increases the duration of gestation and reduces the rate of preterm delivery in rats.^23^ Moreover, blocking allopregnanolone production in late pregnancy leads to preterm labour in rats.^24^

To our knowledge, the GABA_A_ receptor functions in pregnant women with threatened preterm labour (TPL) have not been investigated. We hypothesize that the effect of allopregnanolone via its action on the GABA_A_ receptor is diminished in women with TPL. In this study, we investigated whether circulating levels of progesterone, allopregnanolone and oxytocin differ between women with preterm labour and women with an intact, ongoing pregnancy. We also examined whether the GABA_A_ receptor function, measured with oculography, differs between women who experience preterm labour and women with an intact pregnancy.

## 2 METHODS

Pregnant women with threatened spontaneous preterm labour (patient group) were recruited through the maternity unit at the Department of Obstetrics and Gynaecology, Sundsvall County Hospital, Sundsvall, Sweden. The same number of pregnant women with normal, intact pregnancies (control group) was recruited by advertising among outpatients at the Department of Obstetrics and Gynaecology, Sundsvall County Hospital, and the antenatal care units in Västernorrland County, Sweden.

### 2.1 Inclusion and exclusion criteria

Pregnant women (*n* = 20) aged 18‐40 years with TPL in gestational week 33 + 6 days to 37 + 0 days were included in this study. The control group (*n* = 20) included healthy women with normal, ongoing pregnancies, who were matched for gestational week at inclusion. Preterm labour was defined as labour occurring spontaneously with no obvious cause before gestational week 37 + 0 days. TPL was considered to be established when regular uterine contractions, usually monitored with cardiotocography (CTG), occurred every 3–4 min (duration > 30 s), accompanied by progressive changes in any parameter of the uterine cervix (ie Bishop score) over time.

Women with TPL and a pathological CTG pattern, multiple pregnancies, antepartum haemorrhage, polyhydramniosis, uterine abnormality, congenital foetal abnormality, pre‐eclampsia, diabetes, hypertension, intrauterine growth retardation, premature rupture of membranes, overt maternal infection or active labour (ie ongoing birth) were excluded. Women were also excluded for the following reasons: abnormal eye function, treatment with psychoactive drugs during pregnancy, known psychiatric and/or premenstrual dysphoric disorder before pregnancy, or a history of alcohol and/or drug abuse.

### 2.2 Study design

The screening phase of the study was commenced after the patient had given her written informed consent to participate in the study. Women in the study group were treated for TPL in accordance with the clinical routines of the maternity unit, which stated that patients at gestational week 33 + 6 days or more were not treated with continued tocolysis, but could receive a single or repeated dose of terbutaline (Bricanyl, 0.25 mg; AstraZeneca AB, Sodertälje) by subcutaneous injection after assessment by an obstetrician. Corticosteroid treatment for foetal lung maturation was not considered necessary in this group. Women going into labour were not included in the study for practical reasons. Active labour was defined as regular uterine contractions (≥3 in 10 min, lasting for at least 60 s) together with full effacement (in nulliparous women) and/or cervical dilatation > 3 cm. In patients with an ongoing delivery, the saccadic eye velocity test (SEVT) could not be completed and blood samples could not be taken because of other necessary medical treatments (antibiotics, tocolytic agents, etc), so they were excluded from the study and treated in accordance with the clinical routines in the delivery ward.

In the study group patients, who had not gone into labour, the study procedures included a CTG test (at least 45 min), a speculum examination to detect any bleeding or early membrane rupture, a vaginal examination to determine any cervical changes and an ultrasound examination to assess cervical length, any maternal or foetal anatomic abnormalities, foetal presentation, amniotic fluid volume (single deepest pocket), deviation in foetal weight from reference range estimated by ultrasonography and foetal weight at birth. Routine blood parameters (blood count, C‐reactive protein [CRP], creatinine, alanine aminotransferase [ALT]), bodyweight and height were also measured.

The measurement of saccadic eye velocity (SEV) with oculography is an objective and sensitive way to assess the sensitivity of the GABA_A_ receptor. A saccade is a rapid, jumping movement of the eye from one fixation point to another. Maximal SEV varies between subjects,^25^, ^26^ but is stable within subjects, both within a testing period and between tests. Once a saccade has started, it is outside conscious control and is not subject to motivational influences.^25^, ^26^, ^27^, ^28^, ^29^ SEV is reduced in a dose‐dependent manner by positive modulators of the GABA_A_ receptor, such as benzodiazepines, allopregnanolone and alcohol.^25^, ^30^, ^31^, ^32^, ^33^, ^34^ After the initial examination and evaluation, the study patients underwent SEVT. To avoid interference by circadian variations in allopregnanolone levels, all subjects were tested at the same time of day (the morning after inclusion, at latest). At the beginning of the test, a venous blood sample was drawn to determine the baseline serum concentrations of oxytocin, progesterone and allopregnanolone. GABA_A_ receptor function was measured with SEV (rapid eye movement when the gaze direction is changed), using a saccadometer (Saccadometer Plus, Ober Consulting Sp z o.o.). During testing, after 30‐min rest, the patient sat in a stable chair with her head fixed in a headrest. The same specialist physician performed the measurements in all patients. Direct infrared photo‐oculography was used with visual stimuli 20° apart horizontally. At each time point, data were collected from 30 saccades. The saccade data used for the analysis fulfilled the following pre‐specified criteria: were not rejected by the automatic validation function and had an amplitude of 15°‐25°, duration of <150 ms and latency of >50 ms Data from time points with less than 15 evaluable saccades were regarded as missing. The median SEV at each time point was used for the data analysis. The calculated parameters were latency and peak velocity. The relationship between saccade size and peak velocity is important because it remains constant, even when voluntary control of the saccade is attempted. Each saccade was analysed to determine the peak velocity and latency from the target movement to the onset of the saccade, where reduced peak velocity and increased latency were expected to be induced by allopregnanolone. Saccade velocity is mainly a measurement of central nervous system (CNS) depression, and the latency between target presentation and the onset of movement of a saccade has been widely used to understand the cortical and subcortical aspects of saccade programming.^35^, ^36^ In this study, saccades and peak velocities with amplitudes of 20° were used for the subsequent analysis.

The study was performed in accordance with ethical principles that had their origin in the Declaration of Helsinki, and was approved (Dnr: 2015/248‐31) by the Regional Ethical Review Board of Umeå, Sweden.

### 2.3 Analysis of blood samples

All blood samples were analysed at the same time to reduce the effect of inter‐assay variability. Allopregnanolone levels were measured as previously described.^34^ Briefly, blood samples (0.4 ml) were extracted with diethyl ether (Merck KGaA), and allopregnanolone was separated from the other cross‐reacting steroids by celite column chromatography. Allopregnanolone levels (nmol/L) were measured with a radioimmunoassay (RIA) with rabbit polyclonal antiserum raised against 3α‐hydroxy‐20‐oxo‐5α‐pregnan‐11‐yl‐carboxymethyl‐ether coupled to bovine serum albumin. The sensitivity of the assay was 25 pg. The intra‐ and inter‐assay coefficients of variation (CVs) were 6.5% and 8.5%, respectively.

Progesterone levels (nmol/L) were measured with an automatic immuno‐analyser (Cobas 8000 Module, e602; Roche Diagnostics) at the medical laboratory of Norrland's University Hospital, Umeå, Sweden. The total assay variation was 2.2% (inter‐ and intra‐assay CVs were 1.62% and 1.54%, respectively).

The oxytocin plasma levels (pmol/L) were analysed with a RIA at the Clinical Neurochemistry Laboratory, Sahlgrenska University Hospital, Mölndal, Sweden. Plasma (1000 µl) was centrifuged after it was mixed with 1 mol/L HCl (100 µl), extracted with the standard protocol for Oasis HLB 1cc (30 mg) Extraction Cartridges (Waters), evaporated and redissolved in assay buffer (250 µl; 0.1% bovine serum albumin, 0.005% Tween 80 in phosphate‐buffered saline, pH 7.6). The RIA was performed with duplicates of the calibrators, controls and samples. The calibrators, controls and samples (all 100 µl) were incubated with 50 µl of oxytocin‐directed antiserum at a final dilution of 1:28,000 in assay buffer at 21°C for 24 h. A second incubation in the same manner was performed after the addition of 50 µl of ^125^I‐oxytocin (10,000 cpm) in assay buffer. The free tracer and bound tracer were separated by the addition of 100 µL of anti‐rabbit IgG antibody (AA‐Sac1, IDS), incubation at room temperature for 30 min, the addition of 1 ml of deionized water and centrifugation (2500 *g*, 21°C, 5 min). The supernatant was discarded and the precipitate measured with a gamma counter (1470 Wizard TM Wallac). The intra‐assay CV for the control was 14% at 12 pmol/L oxytocin, 8% at 31 pmol/L oxytocin and 10% at 60 pmol/L oxytocin. The inter‐assay CV for the control was 15% at 12 pmol/L oxytocin, 12% at 32 pmol/L oxytocin and 10% at 58 pmol/L oxytocin.

Routine blood tests, including blood count, CRP and ALT, were analysed at the medical laboratory of Sundsvall County Hospital. The laboratory in Sundsvall is certified by the Swedish Board for Accreditation and Conformity Assessment.

### 2.4 Statistical analysis

All statistical analyses were performed with SPSS version 25 (IBM). Descriptive statistics were used to present the data, which were divided into categorical, ordinal and continuous variables. The normality of the distribution of the data was tested with the Shapiro–Wilk test. Continuous variables were evaluated with the Mann–Whitney *U* test and presented as medians (ranges), whereas categorical and ordinal variables were evaluated with the chi‐square test. The relationships between variables were determined by adjusting for confounders in a multiple logistic regression analysis. Linear regression analyses were performed for continuous variables, presented as unstandardized *B* coefficients and 95% confidence intervals (CIs), and binary logistic regression analyses were performed for categorical variables, presented as odds ratios (ORs), 95% CIs and *p* values. *p* values of <.05 were considered statistically significant.

#### 2.4.1 Power analysis

The results of earlier trials indicated that the standard deviation for SEVT among test individuals was ±7°/s.^34^ The least difference in SEVT between groups that we wanted to detect was 15°/s, which is similar to the results of earlier studies and is considered clinically significant. Therefore, if *α* = .05 and the study power was 90%, the required size of the study population was seven test subjects per group.

## 3 RESULTS

A total of 40 women were enrolled in the study. In the patient group, two patients had used psychoactive drugs (a benzodiazepine or a selective serotonin re‐uptake inhibitor), and in two women, labour progressed rapidly and the examinations and blood tests could not be completed. Therefore, these women were excluded from the study. One patient's blood sample for the progesterone and allopregnanolone tests was haemolysed and could not be analysed, but her results for the other study parameters were included in the statistical analysis. Ultimately, the study group comprised 16 pregnant women with TPL and the control group comprised 20 healthy pregnant women. All patients were followed prospectively until labour commenced.

The baseline characteristics of both groups are shown in Table 1. The number of nulliparous and multiparous women in the TPL group and the control group did not differ significantly (8/8 and 8/12, respectively, *p* > .05). In the TPL group, the length of the uterine cervix and pregnancy duration were shorter than in the healthy women of the control group (*p* < .01 and <.01, respectively). In both groups, gestational age at birth was within the normal range (>37 weeks). A manual examination and CTG recordings showed that the women in the TPL group had more frequent uterine contractions (*p* = .0001) and more often displayed funnelling of the uterine cervix (*p* = .001), but the proportion of patients with a mature cervix (Bishop score > 5) did not differ between the groups. Women with TPL had lower blood CRP than healthy pregnant women (*p* = .016). The two patients whose labour progressed rapidly and were excluded from the study had similar BMI, age and gestational age as patients in the TPL group, but their cervix was mature (Bishop score > 5), and uterine contractions did not decline after tocolysis with terbutaline injections. Therefore, their blood samples and SEVT data were not available.

**Table 1:** Baseline characteristics of women with threatened premature labour (TPL) and controls

	TPL group, *n* = 16		Control group, *n* = 20		*p* Value
	Median	(IQR 1–3)	Median	(IQR 1–3)	
Age (years)	28	27–31.5	27.5	25.3–31	NS
BMI (kg/m^2^)	29.4	25.62–35.30	29.6	25.3–34.7	NS
Blood pressure (mmHg), S/D	120/69	103–132/61–77	120/72	112–129/68–80	NS
Gestational age at enrolment (weeks)	35.8	35.3–36.1	35.1	34.3–35.7	NS
Gestational age at labour (weeks)	38.6	37.4–40.1	40.4	39.5–41.2	.002
dFW (%)	+1	−7.7 to +4.8	+3.8	−6 to +9.4	NS
Amnion water—sDVP (mm)	49	42.8–59.5	53.5	50–67.8	NS
cervical length (cm)	22.5	18–29.5	33	26.3–38.3	.002
Foetal weight (g)	3519	3099–3829	3731	3326–3848	NS
Haemoglobin (g/L)	127.5	115.3–139	121	118–128.3	NS
White blood cells (10^9^/L)	10.5	8.4–13	9	7.6–11.3	NS
CRP (mg/L)	4	2–17	2	0.8–4.5	.016
Creatinine (µmol/L)	52	48–55.8	45.5	41.3–52	NS
ALT (µKat/L)	0.20	0.14–0.30	0.21	0.16–0.30	NS

Abbreviations: ALT, alanine aminotransferase; BMI, body mass index (kg/m^2^); CRP, C‐reactive protein; dFW, deviations in foetal weight from reference range estimated by ultrasonography; IQR, interquartile range; NS, non‐significant; S/D, systolic/diastolic; sDVP, single vertical pocket; W, week.

GABA_A_ receptor function and the sedative effect of allopregnanolone were evaluated with SEVT. The results showed that women in both groups had similar GABA_A_ receptor functions because the saccade latency and peak velocity without external stimulation did not differ between the groups (Table 2).

**Table 2:** Hormone levels and saccadic eye velocity in women with threatened preterm labour and controls

	TPL group		Control group		*p* Value
	Median	IQR (1–3)	Median	IQR (1–3)	
Latency (SEVT) (ms)	164	151–175.5	166.5	158.5–183.5	NS
Peak velocity (SEVT) (degree/s)	527.3	485.6–578.4	551	484.3–647.5	NS
Oxytocin (pmol/L)	41.5	38.3–58.8	37	27.5–45.5	.021
Progesterone (nmol/L)	513	341–681	430	385–536	NS
Allopregnanolone (nmol/L)	49.3	41.5–70	47	38–64	NS
Allopregnanolone/progesterone (ratio)	0.11	0.09–0.13	0.10	0.8–0.13	NS

Abbreviations: IQR: interquartile range; NS, non‐significant; SEVT, saccadic eye velocity test.

In contrast, blood analyses showed that the oxytocin levels were significantly higher among the women with TPL (*p* = .021), although the allopregnanolone and progesterone levels did not differ between the groups. The rate of allopregnanolone conversion from progesterone in the blood, evaluated as the allopregnanolone/progesterone ratio, also did not differ between the groups (Table 2).

The proportion of caesarean deliveries was significantly higher in the TPL group than in the control group (86% vs. 14%, respectively, *p* = .01), and a regression analysis showed that threatened premature delivery was an independent risk factor for caesarean section (OR: 11.4, 95% CI: 1.20–108, *p* = .034). As expected, the oxytocin levels in the blood were associated with preterm labour (OR: 1.09, 95% CI: 1.01–1.2, *p* = .025), whereas the blood levels of progesterone and allopregnanolone were not associated with premature labour. Interestingly, in the TPL group, there was a negative association between blood oxytocin and allopregnanolone (as predictor) levels (*B*: −3.2, 95% CI: −5.5 to −0.9, *p* = .012). Oxytocin was positively associated with progesterone (*B*: 0.353, 95% CI: 0.089–0.617, *p* = .013) and the allopregnanolone/progesterone ratio (*B*: 1.4, 95% CI: 0.12–2.6, *p* = .034), but these associations were not seen in the control group.

When the data were pooled, the overall serum allopregnanolone levels showed a linear correlation with serum progesterone (*r* = .588, *p* = .001) and patients’ body mass index (BMI; *r* = −.414, *p* = .013). BMI as a predictor was associated with lower serum levels of allopregnanolone (*B*: −0.398, 95% CI: −2.53 to −0.256, *p* = .018), but surprisingly, BMI did not correlate with progesterone. There were positive correlations between BMI and oxytocin (*r* = .48, *P* = .034) and between allopregnanolone and progesterone serum levels (*r* = .50, *P* = .024) in the control group only, and these correlations were not seen in the TPL group. However, progesterone as a predictor was associated with the maturity of the cervix (Bishop score; OR: 1.02, 95% CI: 1.00–1.04, *p* = .038) and with a cervical funnelling sign on ultrasound (OR: 1.01, 95% CI: 1.00–1.02, *p* = .017). Neither allopregnanolone nor oxytocin blood levels were related to cervical maturity, but the association between cervical maturity and oxytocin was close to being significant (*p* = .053).

**Figure 2:**
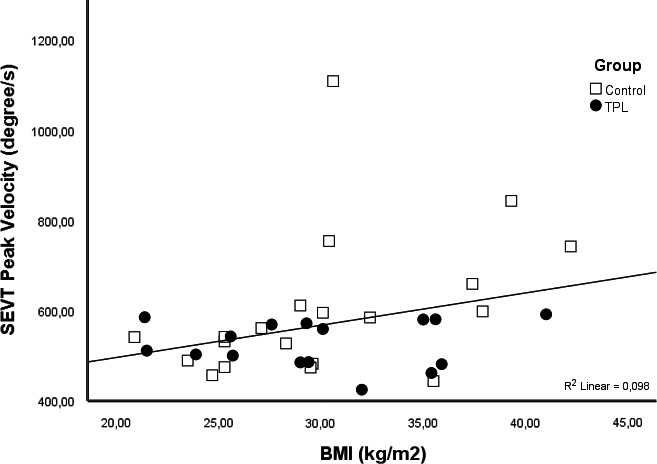
Relationship between SEVT and BMI in the threatened premature labour (TPL) and control groups. Linear regression analysis with fit line at total when data were pooled

In both groups, when the data were pooled, BMI correlated with the saccade peak velocity (*r* = .34, *p* = .044; Figure 2) and negatively with allopregnanolone (*r* = −.41, *p* = .013) and the allopregnanolone/progesterone ratio (*r* = −.35, *p* = .042). There were also correlations between allopregnanolone and progesterone (*r* = .59, *p* < .001) and between allopregnanolone and the allopregnanolone/progesterone ratio (*r* = .64, *p* < .001).

However, when the groups were analysed separately, the saccadic peak velocity correlated with the patients’ BMI only in the control group (*r* = .57, *p* = .009). In women with TPL, the saccadic peak velocity correlated negatively with the cervical length, measured with ultrasound (*r* = −.56, *p* = .025). In both groups, there was a correlation between the serum concentrations of allopregnanolone and progesterone (*r* = .73, *p* = .002 for the TPL group; and *r* = .50, *p* = .024 for the control group). However, the allopregnanolone level was only correlated with the allopregnanolone/progesterone ratio in the control group (*r* = .85, *p* < .001).

## 4 DISCUSSION

In this study, we investigated the involvement of the GABA‐active neurosteroid allopregnanolone and oxytocin in the mechanism underlying TPL.

Our main findings were that women with TPL had higher oxytocin levels in the blood than the control group, whereas their blood levels of allopregnanolone and progesterone were similar. The mean maternal plasma oxytocin level rises gradually in pregnancy towards term, and according to an earlier study, reaches approximately 12.5 µU/ml (~20.1 pmol/L) at term but prior to labour contractions. However, in normal spontaneous labour, no obvious relationship has been observed between the plasma oxytocin levels and uterine contractions,^37^ and blood oxytocin levels vary during different stages of labour.^38^ In rats, premature stimulation by oxytocin secretion can lead to preterm labour, and oxytocin secretion is inhibited by the activation of a central endogenous opioid mechanism in late pregnancy.^39^ In the CNS, allopregnanolone is thought to inhibit the excitation of the oxytocin neurons by inducing an inhibitory opioid mechanism.^21^ Allopregnanolone can be synthesized de novo in the brain, independently of the adrenal glands, placenta and gonads,^40^ and the concentration of allopregnanolone in the brain can be altered independently under different physiological conditions.^41^, ^42^ Allopregnanolone levels correlate with state anxiety during pregnancy, and a reduction in serum allopregnanolone in response to laboratory stressors during the second trimester of human pregnancy has been reported in a pilot study.^43^, ^44^ Potentially stressful conditions during blood sampling, like preterm contractions, could have affected the variations in plasma allopregnanolone and concealed group differences.

The inhibition of oxytocin synthesis by allopregnanolone only occurs in the CNS, and the peripheral blood levels of this neurosteroid may not reflect regional differences in allopregnanolone levels within the brain. Our results show that there was a negative association between the peripheral blood levels of allopregnanolone and oxytocin insofar as when allopregnanolone increased, it reduced blood oxytocin levels. This could indicate that this neurosteroid is, in fact, involved in oxytocin synthesis. We may not have observed the same results in the control group because in the control group, the induction of oxytocin secretion was already physiologically inhibited. We speculate that in normal women, there is a stable equilibrium in the brain that controls oxytocin inhibition through the combined effects of allopregnanolone and the opioid system. However, in women with TPL, this balance is probably compromised, with the consequence that oxytocin concentrations are increased in the blood.

In rats, the administration of a GABA_A_ receptor antagonist and the consequent blockage of allopregnanolone synthesis stimulate oxytocin secretion.^9^, ^45^ However, it is as yet unclear whether the allopregnanolone‐induced inhibitory opioid mechanism is mediated by the GABA_A_ receptor in women. The novelty of this study was using a functional test of the GABA_A_ receptor to investigate whether increased oxytocin blood levels are caused by the reduced central effect of allopregnanolone. Allopregnanolone acts via the GABA_A_ receptor by non‐genomic action, and a dysfunction in the GABAergic system may impair the inhibition of oxytocin synthesis by allopregnanolone.^46^ One method used to measure the functional activation of the GABA_A_ receptor is SEVT. Once a saccade has begun, it is outside conscious control and is not subject to motivational influences. Therefore, SEVT is an objective method of measuring the sedative effect of allopregnanolone and other GABAergic substances.^27^, ^47^ However, there was no difference in the study parameters saccade latency and peak velocity between the TPL and control groups; thus, the GABA_A_ receptor function in patients with TPL did not differ from that in the control group.

Although we know from previous studies that the GABA_A_ receptor function can be more accurately investigated when the receptor function is provoked by exogenous GABAergic substances,^28^, ^29^ in this study we could not use these substances because they might have affected the women during pregnancy, some of whom already displayed TPL. The GABA_A_ receptor converts chemical messages into electrical signals, but before the neurotransmitter GABA is used to stimulate the receptor, only rare brief spontaneous currents are present. Therefore, before any conclusions are drawn, the GABA_A_ receptor function should be measured after the receptor is stimulated with its ligands.

The saccadic peak velocity correlated negatively with the shortening of the uterine cervix. If a shortening of the cervix is a sign of impending premature labour and the peak saccadic velocity reflects the GABA_A_ receptor function, then it implies that in TPL, the GABA_A_ receptor function is downregulated. However, we know that in addition to an indirect effect on the inhibitory opioid mechanism, allopregnanolone also controls oxytocin synthesis by its direct effect on magnocellular oxytocin neurons.^48^ Within the context of other possible mechanisms that increase the inhibition of oxytocin secretion, such as nitrous oxide and phosphorylation‐associated changes in the GABA_A_ receptor function,^49^, ^50^ the interpretation of our findings becomes even more difficult.

Our results suggest that progesterone, but not allopregnanolone, affects the maturity of the cervix. Cervical maturation is a key sign in preterm labour.^51^ Previous attempts to clarify the effect of progesterone on cervical maturation have shown a complex picture, suggesting that progesterone is involved in cervix maturation.^52^, ^53^ In humans, the concentration of progesterone in the myometrium during pregnancy does not differ in patients with different types of labour (induced or spontaneous) or with the contractile activity of the myometrium. Therefore, the expression of the myometrial oxytocin receptor in vivo does not seem to be related to the progesterone concentration in the myometrium.^54^ An earlier in vitro study showed that progesterone reduces the amount of muscular work performed per contraction, but that its metabolite, allopregnanolone, does not have the same effect and does not seem to inhibit the contractions of the human term myometrium. Moreover, none of these substances affect the duration of contractions.^10^ Our results suggest that the peripheral blood level of allopregnanolone did not directly affect the cervix in premature labour, either in the control group or in the TPL group.

We observed that in the combined groups (TPL + control), the patient's saccadic peak velocity correlated with BMI and that blood allopregnanolone levels also correlated with the women's BMI (Figure 2). In pregnant women, allopregnanolone production continues to rise until term and may vary depending on several factors, including gestational age, as well as BMI.^55^, ^56^ Allopregnanolone affects the saccadic peak velocity^57^ and BMI, in turn, affects allopregnanolone production, so a relationship between peak velocity and BMI should be expected.

There were some limitations to this study. A small sample size can compromise a study's capacity to detect clinically relevant differences, but in our study, the number of TPL patients was much higher than the number calculated with the sample size analysis. The method for evaluating the GABA_A_ receptor function was not optimal because the study patients were pregnant, which complicated the situation and prevented us activating the GABA_A_ receptor by challenge with GABAergic substances. Therefore, the results of this study should be confirmed with future studies in which the GABA_A_ receptor function is studied after pharmaceutical challenge. Another possible limitation of the study was that none of the patients in the study group delivered prematurely, despite uterine contractions. Then, the question arises whether both the study group and the control group represent variants of normal pregnancies with temporary contractions in one group. The main purpose in this study, however, was to control allopregnanolone levels and GABA_A_ receptor function when oxytocin levels were increased in the blood. Although women in the study group did not deliver prematurely, they still had contractions and high oxytocin levels in the blood, and hence differed from the control group. Thus, the fact that labour in both groups did not progress has probably not affected the study's results.

In conclusion, our study has shown that the levels of the neurosteroid allopregnanolone were unchanged in the peripheral blood of women with TPL, despite the increase in available oxytocin, but an association between allopregnanolone and oxytocin levels may suggest a central mechanism in which allopregnanolone controls the peripheral oxytocin levels. Although the function of the GABA_A_ receptor was the same in women with and without TPL, SEV should be investigated during challenge with a receptor agonist to ensure reliable results. The physiological mechanisms involved in preterm labour are still incompletely understood, and more research is required to determine the role of progesterone and its metabolite, allopregnanolone, in preterm labour.

## CONFLICTS OF INTEREST

The authors have no conflicts of interest to declare in relation to this article.

## AUTHOR'S CONTRIBUTION

ST and YKF led the cohort and participant recruitment. TB and MB performed neurosteroid analysis, and ST performed data analysis. ST, TB, YKF and MB prepared the manuscript. All authors have approved the final version of this manuscript.

## Data Availability

The data that support the findings of this study are available from the corresponding author upon reasonable request.
